# Supplementary material for: PKC-phosphorylation of Liprin-α3 triggers phase separation and controls presynaptic active zone structure
Source: Nat Commun. 2021 May 24;12:3057. doi: 10.1038/s41467-021-23116-w (PMC8144191; doi:10.1038/s41467-021-23116-w)
Supplement: Supplementary file 1 — Supplementary Information [file 41467_2021_23116_MOESM1_ESM.pdf]

Supplementary Information for Emperador-Melero et al., "PKC-phosphorylation of Liprin-α3 triggers phase separation and controls presynaptic active zone structure"

Supplementary Figures 1 to 10

Supplementary Table 1

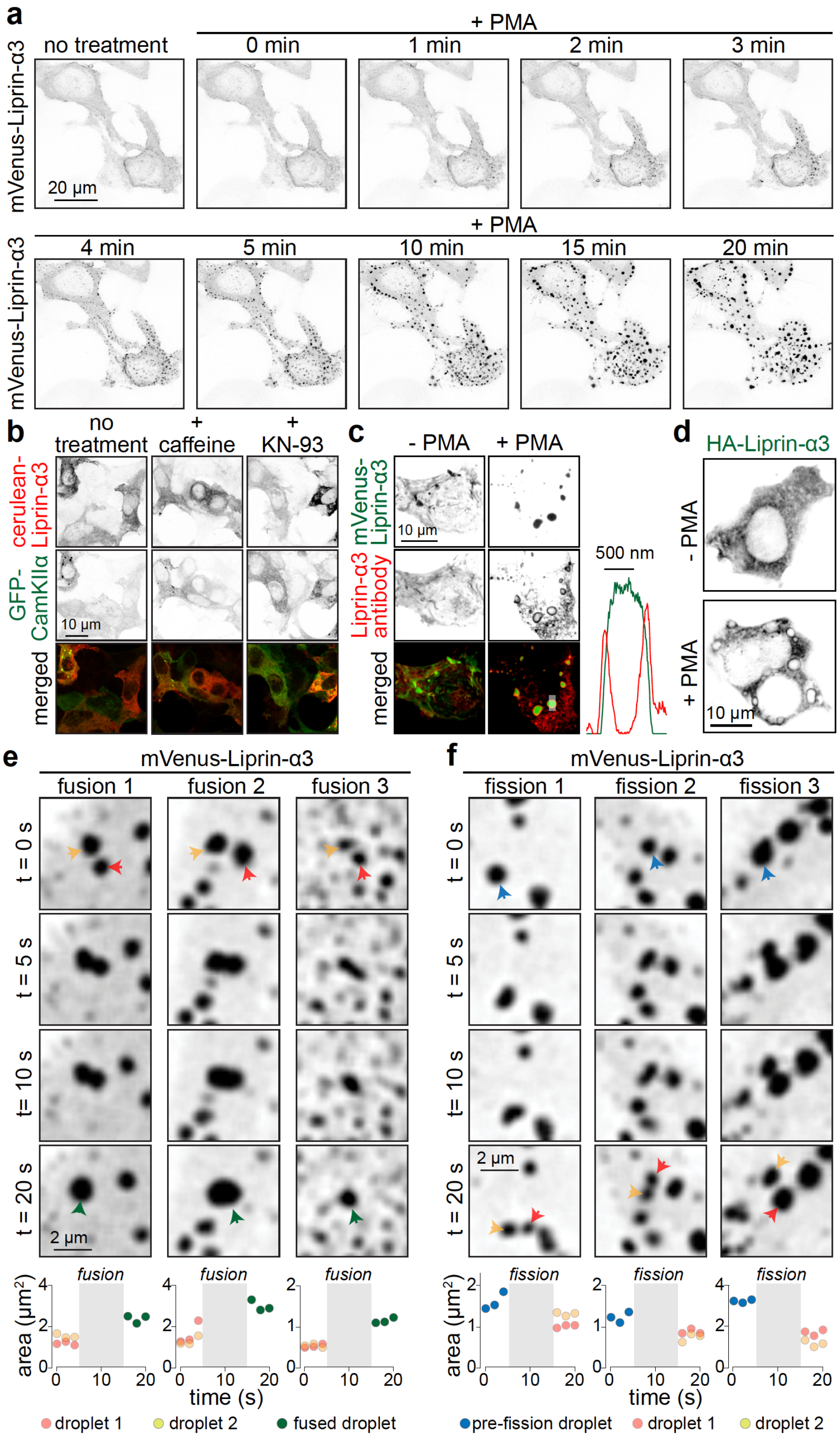

Supplementary Figure 1. Characterization of Liprin-α3 droplet formation, and of condensate fusion and fission

(a) Example confocal time-lapse images of live HEK293T cells transfected with mVenus-Liprin-

α3 during PMA addition; also see Supplementary Movie 1, example images from one of two time-lapse movies are shown.

(b) Example confocal images of fixed HEK293T cells transfected with cerulean-Liprin-α3 and GFP-CamKIIα, without drug treatment or in the presence of caffeine (CamKII activator) or KN-93 (CamKII inhibitor). No formation of Liprin-α3 condensates was observed. In some cells, CamKIIα was present in dense, droplet-like compartments, but these did not contain Liprin-α3, representative cells from 2 images (containing several cells for each condition, 1 transfection per condition) are shown.

(c) Example confocal images of fixed HEK293T cells transfected with mVenus-Liprin-α3 and immunostained for Liprin-α3. A line profile of a Liprin-α3 condensate is shown on the right. Note that antibody staining produces ring-like shapes around mVenus-Liprin-α3 fluorescence, likely because antibodies do not enter the fixed phase condensates, representative cells from 2 images (containing several cells for each condition, 1 transfection per condition) are shown.

(d) Example confocal images of fixed HEK293T cells transfected with HA-Liprin-α3 and immunostained for HA. Ring-like structures similar to antibody-stained mVenus-Liprin-α3 in d were present after PMA addition, indicating that phase condensation of Liprin-α3 is independent of the presence of a fluorescent tag, representative cells from 2 images (containing several cells for each condition, 1 transfection per condition) are shown.

(e, f) Time-lapse confocal images of three mVenus-Liprin-α3 droplets each undergoing fusion (e) or fission (f) in live HEK293T cells. Plots of the droplet area before and after the fusion/fission events are shown below the images, three fusion and three fission events were analyzed for each condition from two independent time-lapse movies.

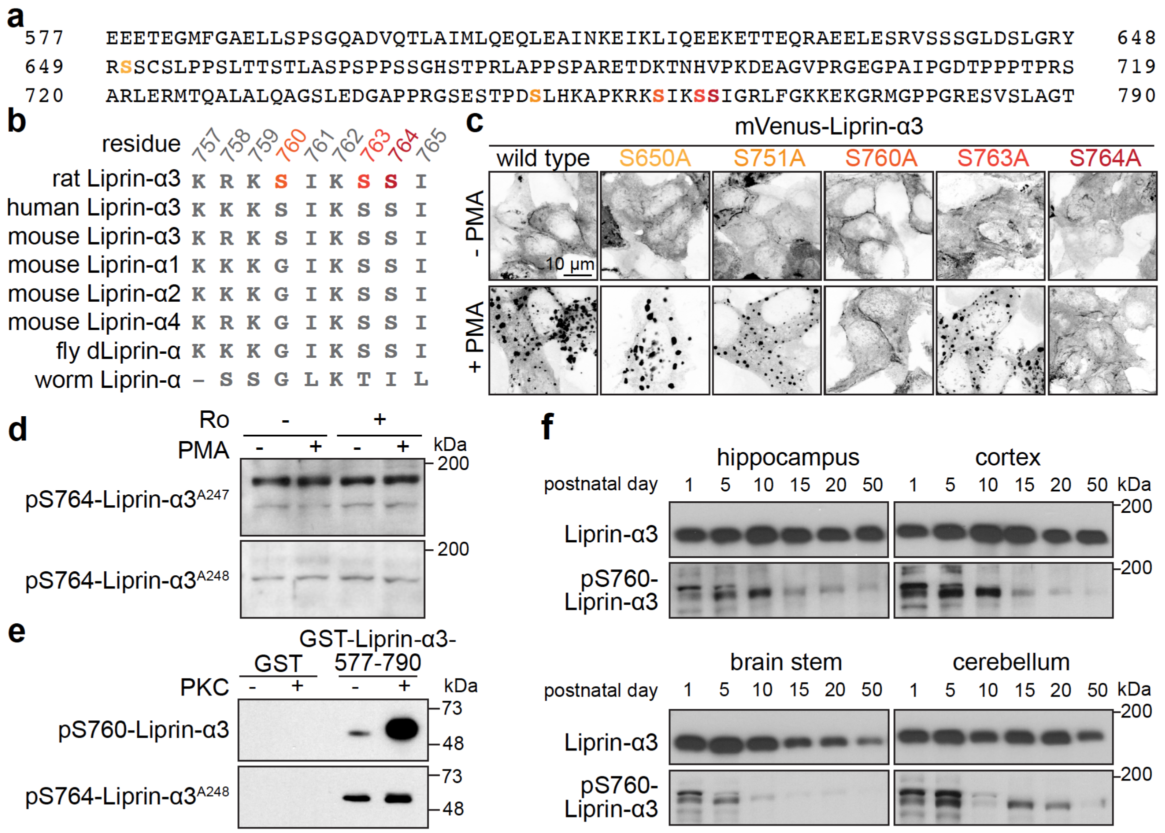

Supplementary Figure 2. Characterization of Liprin-α3 PKC phosphorylation in vitro and in vivo

(a) Amino acid sequence of the Liprin-α3 fragment that was highly phosphorylated. The serines identified by phospho-proteomic analyses are highlighted in a color code repeated in b and c.

(b) Amino acid sequences of various Liprin-α isoforms around S760. The PKC phosphorylation site is conserved in Liprin-α3 among vertebrates, but absent in Liprin-α1, -α2 and -α4, and in the Liprin-α proteins expressed in *C. elegans* and *D. melanogaster*.

(c) Confocal images of fixed HEK293T cells transfected with mVenus-tagged Liprin-α3 or mVenus-Liprin-α3 containing point mutations of each candidate amino acid residue potentially phosphorylated by PKC. Note that S760A and S764A abolish condensate formation upon PMA addition. All constructs contained single point mutations, except for the S650A construct, which also contained Y648A and S651A point mutations, representative cells from 2 images (containing several cells for each condition, 1 transfection per condition) are shown.

(d) Western blot of HEK293T cell lysates transfected with mVenus-Liprin-α3 showing that activating or blocking PKC phosphorylation (with PMA and Ro31-8220, respectively) does not change the signal detected by phospo-S764 specific Liprin-α3 antibodies (two independent antisera raised against the same epitope in different rabbits – A247 and A248 – are shown), and hence S764 is unlikely a substrate of PKC. This experiment was run at the same time as the experiment with pS760-Liprin-α3 antibodies shown in Fig. 2c, and the Liprin-α3 control blot shown in Fig. 2c is valid for this figure as well, representative scans from two independent western blots are shown.

(e) Western blot of purified GST-Liprin-α3-577-790 or GST alone in the presence or absence of recombinant PKC. Note that phospo-S764 (A248) specific Liprin-α3 antibodies do not detect a signal increase after PKC addition, while phospo-S760 specific Liprin-α3 antibodies do. Loading was assessed qualitatively by Ponceau staining of the membrane before antibody incubation and was similar, representative scans from two independent western blots are shown.

(f) Western blots showing the expression profile of phospho-S760 Liprin-α3 across brain areas and development. Note the high expression levels during early postnatal days and synaptogenesis, representative scans from two independent western blots are shown.

Source data for d-f are provided in the Source Data file.

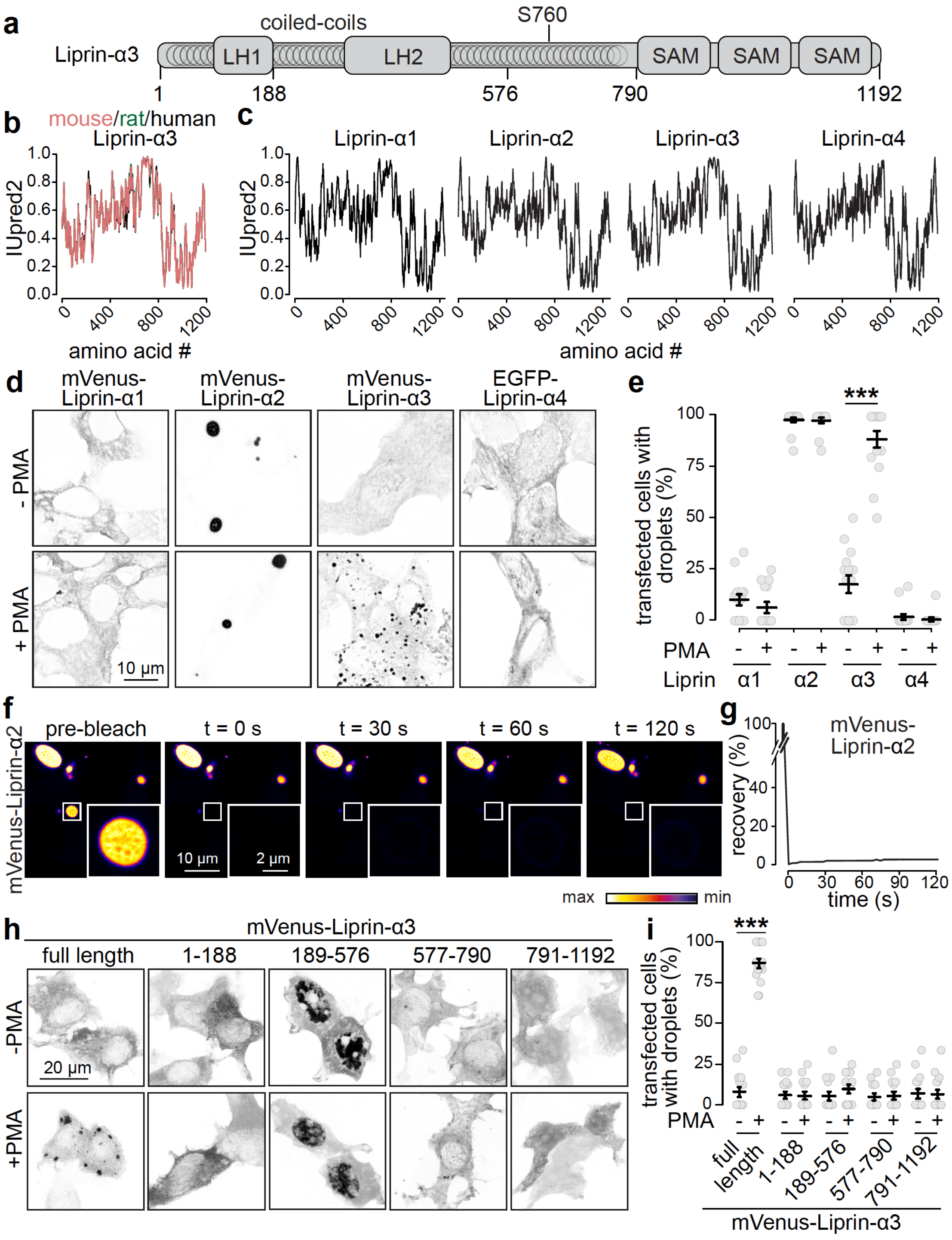

Supplementary Figure 3. Additional characterization of Liprin-α phase separation

(a) Schematic of the Liprin-α3 domain structure showing Liprin homology regions 1 and 2 (LH-1 and -2), coiled-coil regions and sterile alpha motifs (SAM).

(b, c) Assessment of the presence of intrinsically disordered regions (IDRs) predicted by

IUpred2 [33] for human, mouse and rat Liprin-α3 (b), and for each individual mouse Liprin-α (c). Higher IUpred2 scores indicate intrinsically disorganized regions based on context-dependent predictions of protein disorder.

(d, e) Example confocal images (d) and quantification (e) of fixed HEK293T cells transfected with mVenus-tagged Liprin-α1, -α2, and -α3 or EFGP-tagged Liprin-α4 with or without PMA. Of note, only Liprin-α3 forms condensates in the presence of PMA, while Liprin-α2 forms them constitutively and Liprin-α1 and -α4 do not frequently form such structures. N = 15 images/3 independent transfections per condition, p-values: Liprin-α1, 1.0; Liprin-α2, 1.0; Liprin-α3, 0.00004 (***), Liprin-α4, 1.0.

(f, g) Example time-lapse images (f) and quantification (g) of FRAP of mVenus-Liprin-α2 condensates in live, transfected HEK293T cells. Note the absence of fluorescence recovery despite the round, droplet-like appearance. N = 12 droplets/3 independent transfections.

(h, i) Example confocal images (h) and quantification (i) of fixed HEK293T cells transfected with mVenus-tagged Liprin-α3 fragments or full-length Liprin-α3 in the presence or absence of PMA. Note that no individual fragment forms condensates either with or without PMA. N = 15 images/3 independent transfections per condition, p-values: full length, 0.00009 (***);1-188, 1.0; 189-576, 1.0; 577-790, 1.0; 791-1192, 1.0.

Data in e, g and i are plotted as mean ± SEM. Significance was assessed by Kruskal-Wallis tests with Holm post-hoc comparisons between all groups, and only results compared to the corresponding - PMA condition are reported. All tests were two-sided.

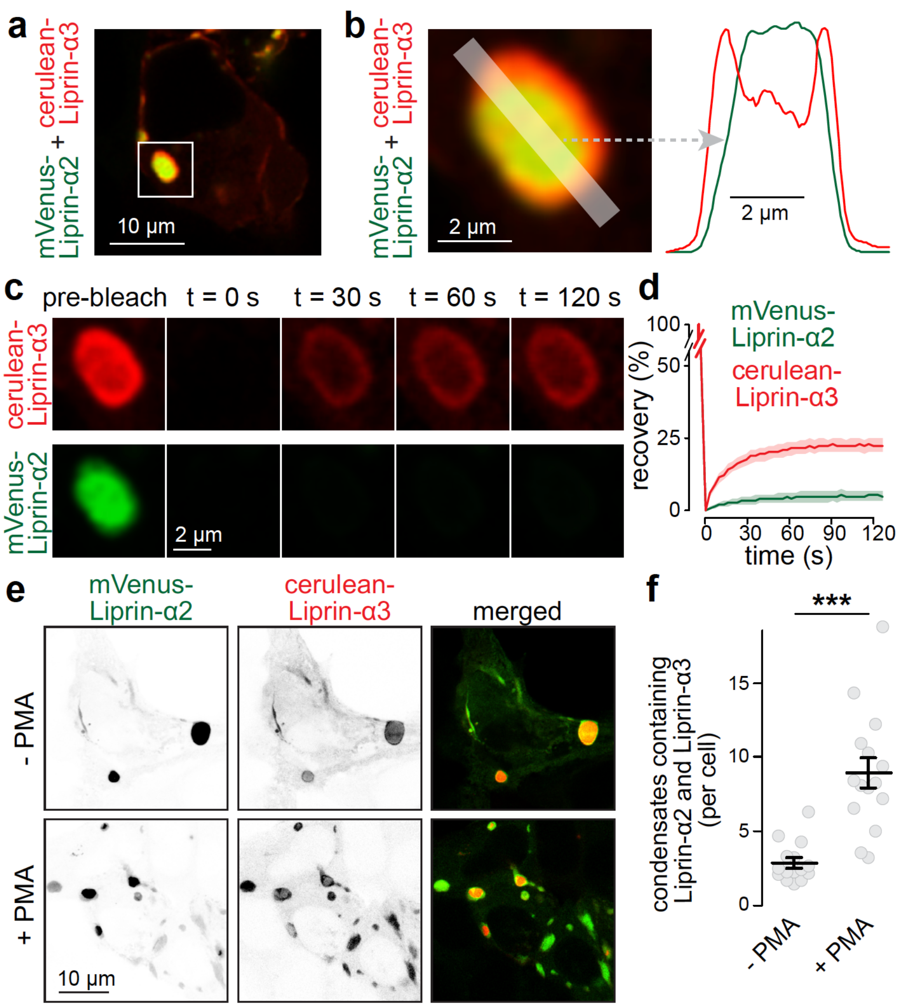

Supplementary Figure 4. Characterization of condensates containing Liprin-α2 and Liprin-α3

(a, b) Example confocal image of a live HEK293T cell transfected with mVenus-Liprin-α2 and cerulean-Liprin-α3 (a), and zoom-in of a condensate and line profile perpendicular to the shorter axis of the condensate (b). Note that Liprin-α3 localizes to the periphery of the condensate, while Liprin-α2 localizes to the center, representative condensate from 2 images (containing several cells, 1 transfection) are shown.

(c, d) Time-lapse images (c) and quantification (d) of the fluorescence recovery after photobleaching of the condensate shown in b. Note the fluorescence recovery of cerulean-Liprin-α3, but not of mVenus-Liprin-α2. N = 15 droplets/3 independent transfections.

(e, f) Example confocal image (e) and quantification of the number of condensates containing Liprin-α2 and Liprin-α3 per cell (f) in fixed HEK293T cells transfected with mVenus-Liprin-α2 and cerulean-Liprin-α3 with or without of PMA. N = 15 images/3 independent transfections per condition, p-value: 9e-07 (***).

Data in d and f are mean ± SEM. Significance was assessed by a two-sided Mann-Whitney U test in f.

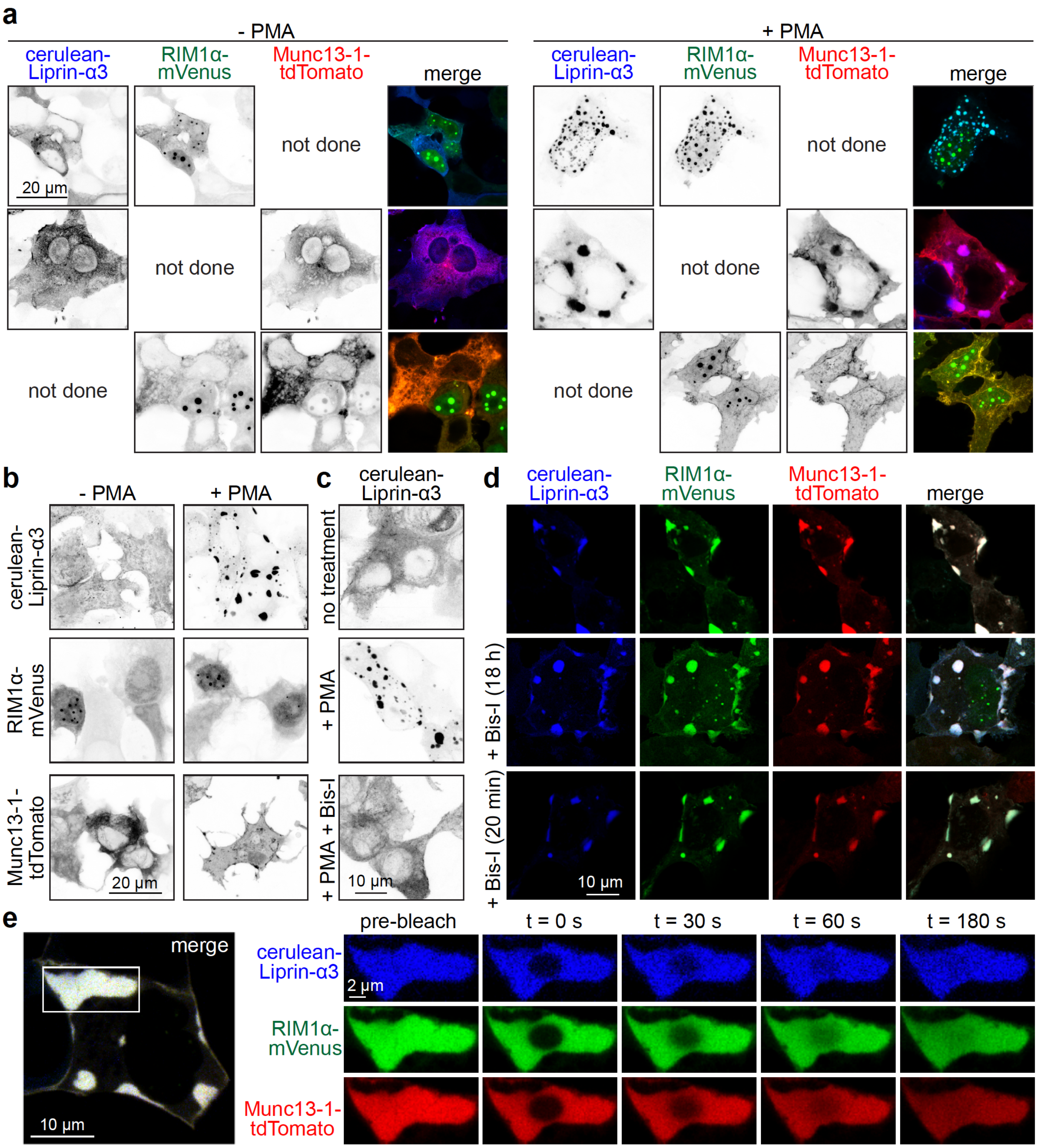

Supplementary Figure 5. Properties of droplets formed between Liprin-α3, RIM1α and Munc13-1

(a, b) Example confocal images of HEK293T cells transfected with combinations of two cDNAs of cerulean-Liprin-α3, RIM1α-mVenus and Munc13-1-tdTomato (a) or with only one cDNA (b) in the presence or absence of PMA. Note that PMA only increases formation of large droplet-like

condensates when Liprin-α3 is expressed, representative cells from 2 images (containing several cells for each condition, 1 transfection per condition) are shown.

(c) Example confocal images of fixed HEK293T cells transfected with cerulean-Liprin-α3 and incubated in the presence or absence of PMA, and in the presence of PMA and the PKC inhibitor Bis-I. Bis-I was added one hour before PMA, representative cells from 2 images (containing several cells for each condition, 1 transfection per condition) are shown.

(d) Example confocal images of fixed HEK293T cells transfected with cerulean-Liprin-α3, RIM1α-mVenus and Munc13-1-tdTomato incubated with the PKC inhibitor Bis-I either at the time of transfection (cells fixed 18 h after transfection) or 20 minutes before fixation, representative cells from 1 image per condition (containing several cells for each condition, 1 transfection) are shown.

(e) Example FRAP experiment of a membrane-proximal condensate containing cerulean-Liprin-α3, RIM1α-mVenus and Munc13-1-tdTomato imaged in transfected, live HEK293T cells in which only the center of the large condensate was photo-bleached (a different condensate from the cell in Fig. 3f is shown). Note the fast recovery of all three proteins, indicative of active internal protein rearrangement, for number of repeats, see Figs. 3f, 3g.

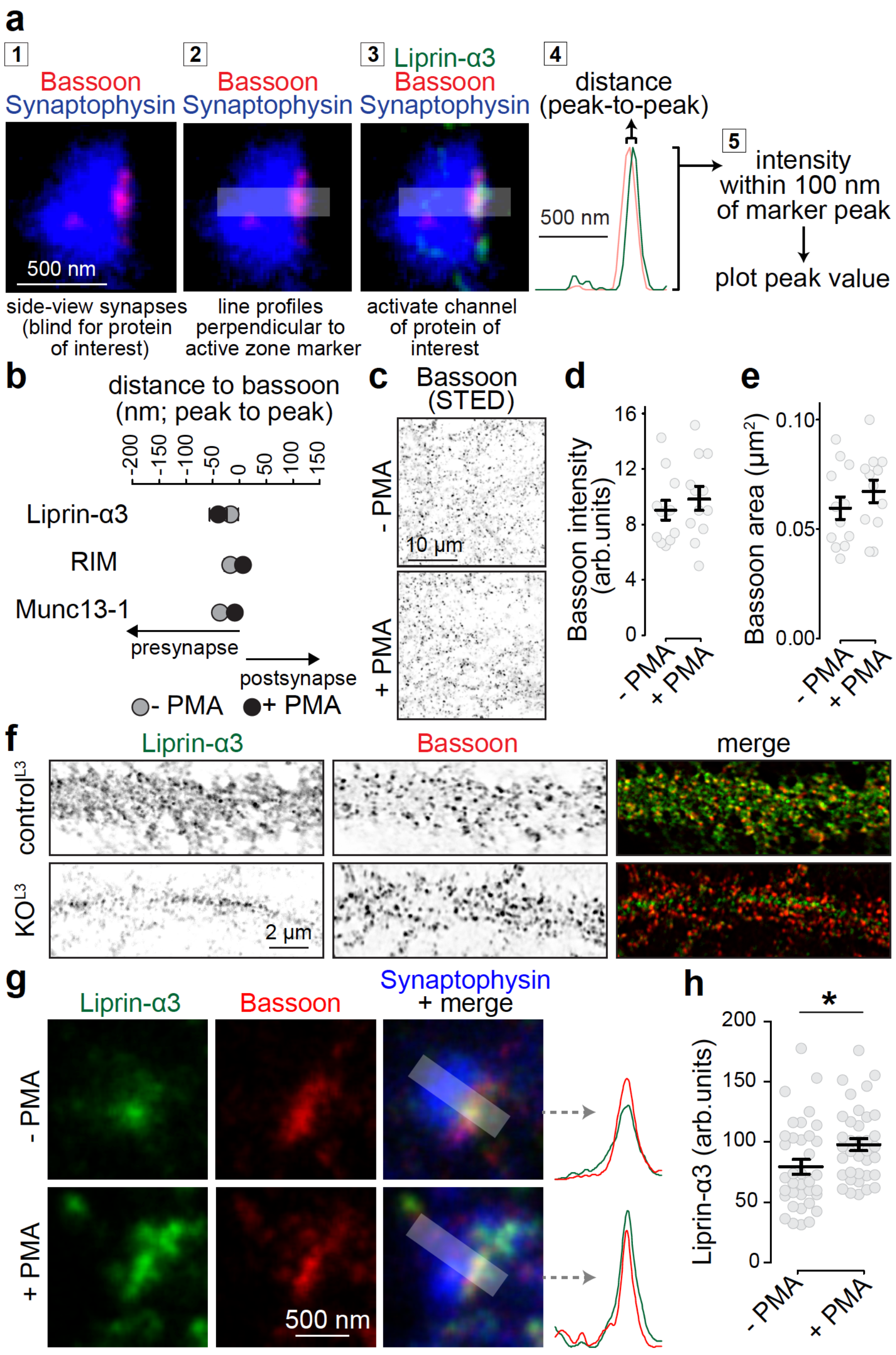

Supplementary Figure 6. STED side-view synapse analysis workflow, and assessment of peak position of active zone proteins and of Liprin-α3 with a newly generated antibody

(a) Data analyses workflow for STED side-view synapses, showing an example STED image of a wild type side-view synapse immunostained for Bassoon and Liprin-α3 (imaged in STED mode) and Synaptophysin (imaged in confocal mode).

(b) Quantification of the average distance of the peak of Liprin-α3, RIM and Munc13-1 of the experiment shown in Figs. 3i-3j to the peak of Bassoon. Liprin-α3: N = 71 synapses/3 independent cultures (- PMA) and 65/3 (+ PMA); RIM: N = 55/3 (-PMA) and 54/3 (+ PMA); Munc13-1 N = 46/3 (- PMA) and 44/3 (+ PMA), p-values: Liprin-α3, 0.12; RIM, 0.32; Munc13-1, 0.07.

(c-e) Example images (c) and quantification of the average intensity (d) and size (e) of Bassoon objects detected using automatic two-dimensional segmentation (size filter of 0.04-0.4 μm^2 without considering the shape or orientation of the signal), N = 12 images/3 cultures per condition, p-values: d, 0.55; e, 0.31.

(f) Example confocal images of Liprin-α3 control ($control^{L3}$) and knockout (KO^{L3}) neurons immunostained with a newly generated antibody (A232). Note that the punctate staining was removed in KO^{L3} neurons, confirming antibody specificity, a representative magnified area from an overview image is shown, 5 overview images were taken for each condition.

(g, h) Example STED images and line profiles (g) and quantification of peak intensities (h) of Liprin-α3 (using the A232 antibody, different from the one used in Fig. 3i, 3j, see panel f) at Synaptophysin positive side-view synapses identified via Bassoon. N = 37 synapses/3 independent cultures (-PMA) and N = 36/3 (+PMA), p-value: 0.02 (*).

Data are shown as mean ± SEM. Significance was assessed by Mann-Whitney rank sum tests (b, h) or t-tests (d, e). All tests were two-sided.

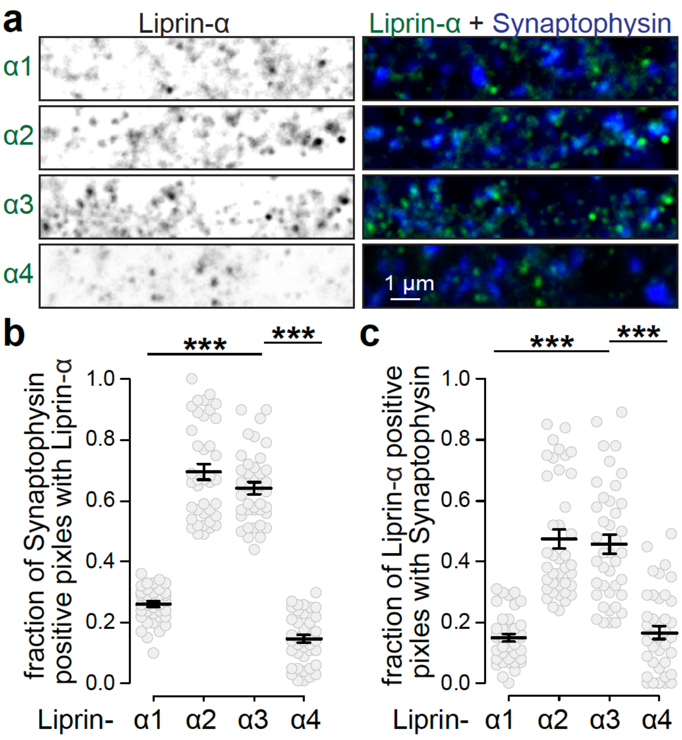

Supplementary Figure 7. Quantification of Liprin-α proteins at synapses

(a) Confocal images of mouse hippocampal cultured neurons stained for Synaptophysin and Liprin-α1, -α2, -α3 or -α4. For knockout-validation of Liprin-α2 and Liprin-α3 antibodies, see Figs 4b, 4c, Supplementary Fig. 8e and [19], for validation of Liprin-α1 and Liprin-α4 antibodies, see [34].

(b, c) Mander's correlation for the fraction of Synaptophysin pixels positive for Liprin-α1, -α2, -α3 or -α4 (b), and vice versa (c). N = 39 images/3 independent cultures for Liprin-α1, -α2; N = 40/3 for Liprin-α3, -α4, p-values (compared to Liprin-α3): b, Liprin-α1, 1e-13 (***), Liprin-α2, 0.21, Liprin-α4, 9e-14 (***); c, Liprin-α1, 2e-12 (***), Liprin-α2, 1.0, Liprin-α4, 3e-09 (***).

Data are shown as mean ± SEM. Significance was assessed by Kruskal-Wallis test followed by Holm post-hoc comparison against Liprin-α3. All tests were two-sided.

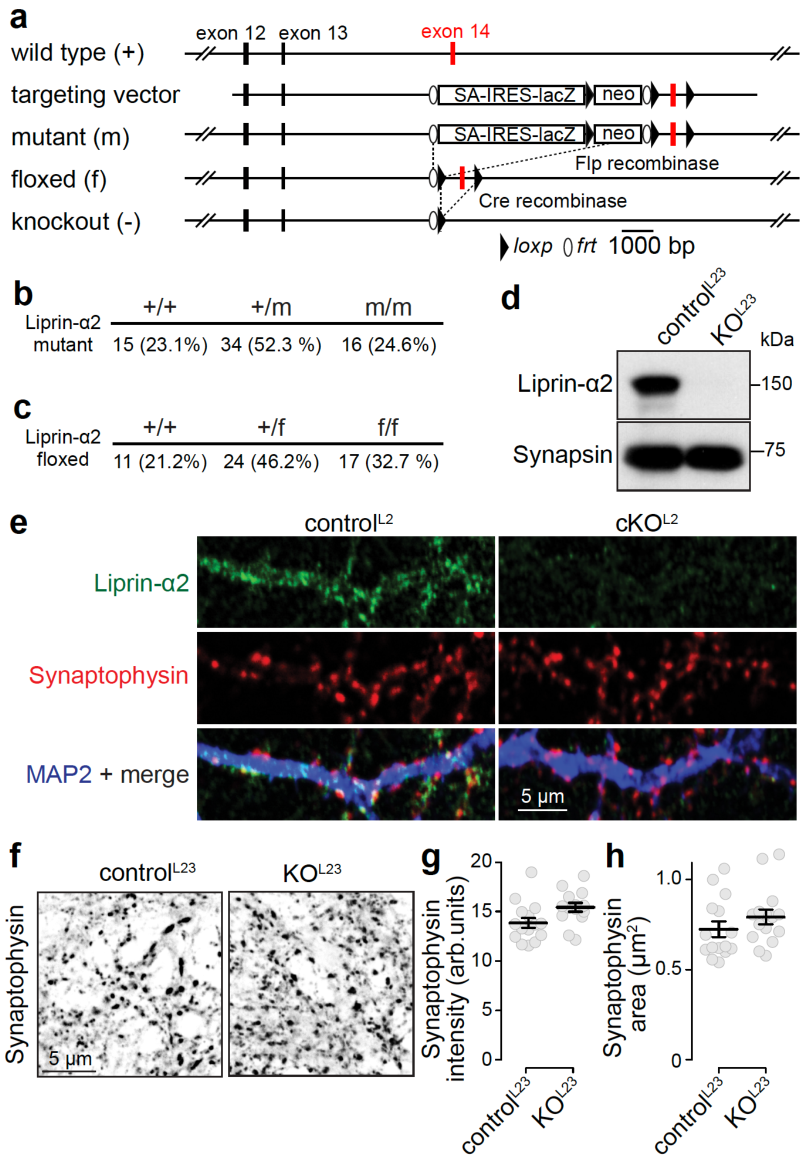

Supplementary Figure 8. Generation of conditional Liprin-α2 knockout mice

(a) Diagram outlining the gene targeting experiment [69]. The targeting vector contained exon 14 flanked by *loxP* sites, and splice-acceptor/lacZ and neomycin resistance cassettes flanked by *frt* sites. Homologous recombination in embryonic stem cells resulted in the Liprin-α2 mutant allele, and breeding to flp-transgenic mice [70] was used to produce the floxed allele. Cre recombinase can then be used to generate the knockout allele.

(b, c) Survival ratios of the original mutant (b) and floxed (c) alleles, the total number (and the percent of total) of offsprings of heterozygote matings are shown, p-values (compared to

expected offspring numbers according to Mendelian inheritance): b, 0.92; c, 0.43.

(d) Western blot of cultured control^{L23} and cKO^{L23} neurons. Liprin-α2 was efficiently removed, a representative scan of 9 independent experiments is shown.

(e) Immunostaining of cultured neurons from Liprin-α2 floxed mice that were infected with lentiviruses that express Cre recombinase (to generate cKO^{L2} neurons) or with lentiviruses that express a recombination deficient truncation of Cre (to generate control^{L2} neurons). Liprin-α2 was efficiently removed upon Cre recombination, a representative magnified area from an overview image from one culture is shown.

(f-h) Example images (f) and quantification of the average intensity (g) and size (h) of Synaptophysin objects detected using automatic two-dimensional segmentation (size filter of 0.5-5 μm^2 without considering the shape or orientation of the signal), N = 15 images/3 cultures per condition, p-values: g, 0.08, h, 0.07.

Data are shown as mean ± SEM. Significance was assessed by Chi-square tests (b, c), a t-test (g) or a Mann-Whitney rank sum test (h). All tests were two-sided. Source data for d are provided in the Source Data file.

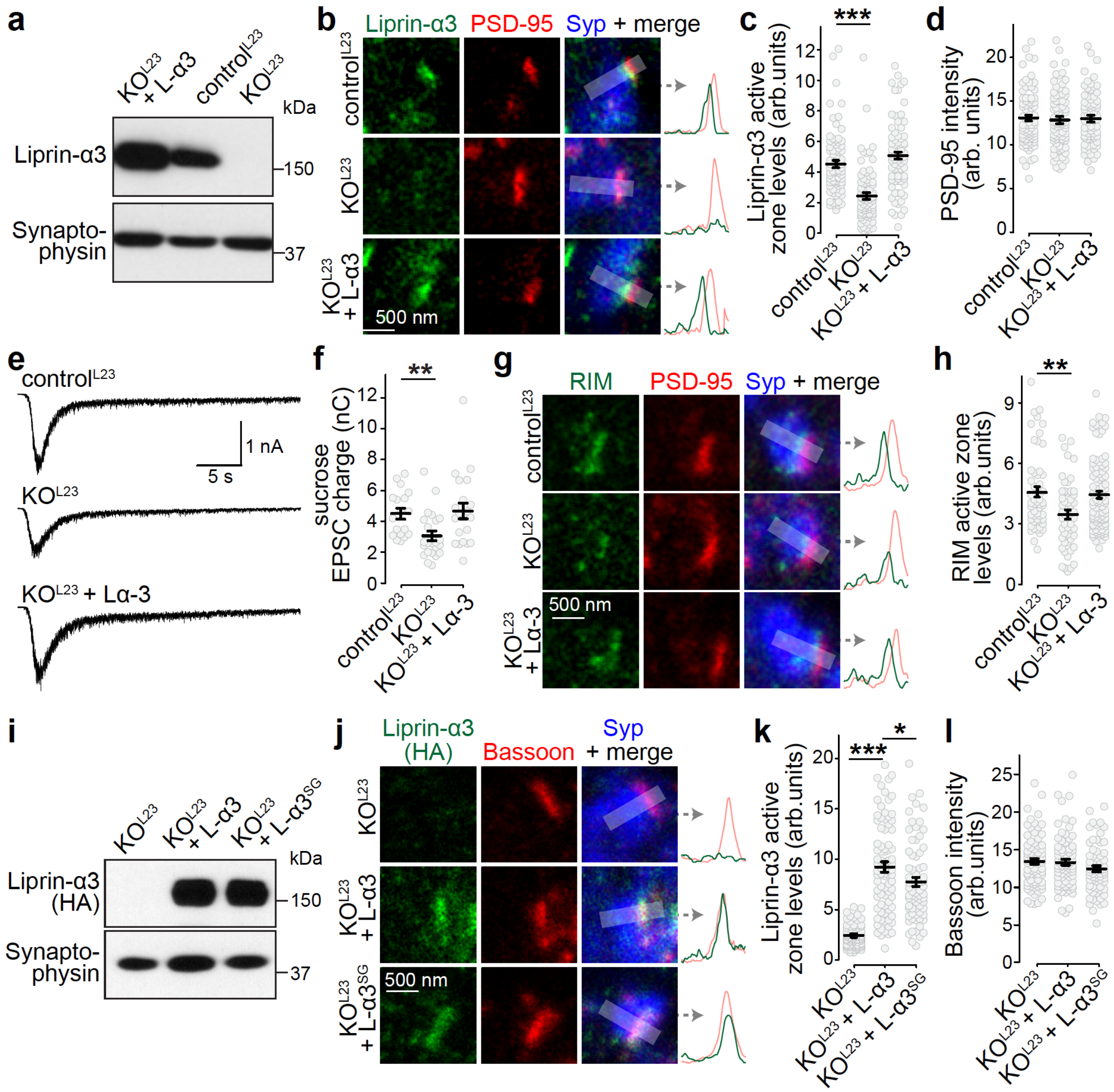

Supplementary Figure 9. Rescue with Liprin-α3 reverses Liprin-α2/α3 knockout phenotypes

(a) Western blot of whole cell lysates of control^{L23}, KO^{L23} and KO^{L23} rescued with Liprin-α3 (KO^{L23} + L-α3) neuronal cultures, a representative scan of 3 independent experiments is shown.

(b-d) Example STED images with intensity profiles (b) and quantification (c, d) of the peak intensities of Liprin-α3 and PSD-95 in side-view synapses, control^{L23}: N = 83 synapses/3 independent cultures, KO^{L23}: N = 82/3, KO^{L23} + L-α3: N = 77/3, p-values (against control^{L23}): c, KO^{L23}, 2e-12 (***), KO^{L23} + L-α3, 0.07; d, 0.93.

(e, f) Example traces (e) and quantification (f) of EPSC charge in response to a local 10 s puff of 500 mOsm sucrose to estimate the RRP, $control^{L23}$: N = 18 cells/3 independent cultures, KO^{L23}: N = 24/3, KO^{L23} + L-α3: N = 21/3, p-values (compared to $control^{L23}$): KO^{L23}, 0.004 (**), KO^{L23} + L-α3, 0.70.

(g, h) Example STED images with intensity profiles (g) and quantification (h) of the peak intensity of RIM at the active zone of side-view synapses, $control^{L23}$: N = 54/3, KO^{L23}: N = 54/3, KO^{L23} + L-α3: N = 97/3. p-values (compared to $control^{L23}$): KO^{L23}, 0.001 (**), KO^{L23} + L-α3, 0.36.

(i) Western blot of whole cell lysates of KO^{L23}, KO^{L23} + L-α3 or KO^{L23} + L-$α3^{SG}$ neuronal cultures. An antibody against the HA tag was used for detection, a representative scan of 3 independent experiments is shown.

(j-l) Representative STED images with intensity profiles (j) and quantification (k, l) of the peak intensities of Liprin-α3 (using an HA antibody) and Bassoon in side-view synapses, KO^{L23}: N = 81/3, KO^{L23} + L-α3: N = 86/3, KO^{L23} + L-$α3^{SG}$: N = 75/3, p-values: k (compared to KO^{L23} + L-α3) KO^{L23} 2e-16 (***), KO^{L23} + L-$α3^{SG}$ 0.04; l, 0.62.

All data are shown as mean ± SEM. Significance was assessed by one-way ANOVA tests (d, l), or Kruskal-Wallis followed by a posthoc Holm tests against all groups, but only results against $control^{L23}$ are reported in c, f and h, and against KO^{L23} + L-α3 in k. All tests were two-sided. Source data for a and i are provided in the Source Data file.

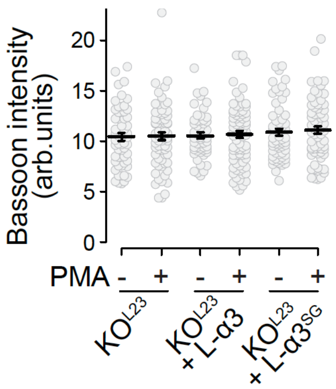

Supplementary Figure 10. Bassoon levels after re-expression of Liprin-α3 and addition of PMA

Quantification of the peak intensity of Bassoon in the data set shown in Fig. 8B. KO^{L23}: N = 60 synapses/3 independent cultures (- PMA) and 71/3 (+ PMA), KO^{L23} + L-α3: N = 54/3 (- PMA) and 83/3 (+ PMA); KO^{L23} + L-$\alpha3^{SG}$: N = 63/3 (- PMA) and 68/3 (+ PMA), p-value: 0.86.

Data are shown as mean ± SEM and analyzed using a two-sided Kruskal-Wallis test.

Genotyping reaction	Primer name	Primer sequence
Ppfia2 wild type allele	G55	GCCTCTTAACATTCACTGTACC
	G56	CCAGTGTGTACTGGAGACAAGC
Ppfia2 mutant allele	G55	GCCTCTTAACATTCACTGTACC
	G57	CTGCGACTATAGAGATATCAACC
Ppfia3 wild type allele	G58	AGAGCCGGCCTATGGACGAC
	G59	GATCTTCAAGCCAGTTCTGC
Ppfia3 mutant allele	G59	GATCTTCAAGCCAGTTCTGC
	G60	GAGCCGGCCTATGGATGCTC

Supplementary Table 1. Genotyping oligonucleotides.

Primer pairs used to genotype Liprin-α2 (*Ppfia2*) mutant mice and Liprin-α3 (*Ppfia3*) mutant mice are shown.
