# Supplementary material for: PKC-phosphorylation of Liprin-α3 triggers phase separation and controls presynaptic active zone structure
Source: Nat Commun. 2021 May 24;12:3057. doi: 10.1038/s41467-021-23116-w (PMC8144191; doi:10.1038/s41467-021-23116-w)
Supplement: Supplementary file 3 — Description of Additional Supplementary Files [file 41467_2021_23116_MOESM3_ESM.docx]

Description of Additional Supplementary Files

Title: Supplementary Movie 1.

Description: Liprin-α3 forms droplets within minutes after addition of PMA Time-lapse confocal movie showing formation of liquid condensates in live HEK293T cells transfected with mVenus-Liprin-α3 upon PMA addition. Black boxes highlight two examples of fusion reactions between condensates. Note that condensates are mobile, in agreement with liquid dynamics, total time of experiment is 20 min compressed to 10 s. One example cell from two movies containing several cells each is shown.
