# Supplementary material for: PKC-phosphorylation of Liprin-α3 triggers phase separation and controls presynaptic active zone structure
Source: Nat Commun. 2021 May 24;12:3057. doi: 10.1038/s41467-021-23116-w (PMC8144191; doi:10.1038/s41467-021-23116-w)
Supplement: Supplementary file 5 — Reporting Summary [file 41467_2021_23116_MOESM5_ESM.pdf]

nature research

Corresponding author(s): Pascal Kaeser

Last updated by author(s): Mar 25, 2021

Reporting Summary

Nature Research wishes to improve the reproducibility of the work that we publish. This form provides structure for consistency and transparency in reporting. For further information on Nature Research policies, see our Editorial Policies and the Editorial Policy Checklist.

Statistics

For all statistical analyses, confirm that the following items are present in the figure legend, table legend, main text, or Methods section.

n/a	Confirmed	
☐	☒	The exact sample size (*n*) for each experimental group/condition, given as a discrete number and unit of measurement
☒	☐	A statement on whether measurements were taken from distinct samples or whether the same sample was measured repeatedly
☐	☒	The statistical test(s) used AND whether they are one- or two-sided<br>*Only common tests should be described solely by name; describe more complex techniques in the Methods section.*
☒	☐	A description of all covariates tested
☐	☒	A description of any assumptions or corrections, such as tests of normality and adjustment for multiple comparisons
☐	☒	A full description of the statistical parameters including central tendency (e.g. means) or other basic estimates (e.g. regression coefficient) AND variation (e.g. standard deviation) or associated estimates of uncertainty (e.g. confidence intervals)
☐	☒	For null hypothesis testing, the test statistic (e.g. *F*, *t*, *r*) with confidence intervals, effect sizes, degrees of freedom and *P* value noted<br>*Give P values as exact values whenever suitable.*
☒	☐	For Bayesian analysis, information on the choice of priors and Markov chain Monte Carlo settings
☒	☐	For hierarchical and complex designs, identification of the appropriate level for tests and full reporting of outcomes
☒	☐	Estimates of effect sizes (e.g. Cohen's *d*, Pearson's *r*), indicating how they were calculated

Our web collection on statistics for biologists contains articles on many of the points above.

Software and code

Policy information about availability of computer code

Data collection	Commercially available software was used for data collection, including pClamp 10.5 for electrophysiological recordings.
Data analysis	Commercially available software and ImageJ were used for data analyses, including Image J Fiji (1.52j99/Java version 1.8.0_66), Matlab (R2020a), R (3.6.3), GraphPad Prism (8.4.1) and IUpred2A. A custom MatLab code for automatic two-dimensional segmentation that was used for detection of Bassoon objects, was previously published (PMID32616470, PMID29398114), and is available on Github (https://github.com/hmslcl/3D_SIM_analysis_HMS_Kaeser-lab_CL and https://github.com/hmslcl/3D_SIM_analysis_HMS_Kaeser-lab_CL).

For manuscripts utilizing custom algorithms or software that are central to the research but not yet described in published literature, software must be made available to editors and reviewers. We strongly encourage code deposition in a community repository (e.g. GitHub). See the Nature Research guidelines for submitting code & software for further information.

Data

Policy information about availability of data

All manuscripts must include a data availability statement. This statement should provide the following information, where applicable:

- Accession codes, unique identifiers, or web links for publicly available datasets
- A list of figures that have associated raw data
- A description of any restrictions on data availability

All analyzed data are included in the figures. Original data generated in the current study are available from the corresponding author on reasonable request. NM_001270985.2 was used to determine the amino acid position of Liprin-α3. For IDR analyses, the following UniProt IDs were used: P60469 (mouse Liprin-α3), Q91Z79 (rat Liprin-α3), O75145 (human Liprin-α3), B2RXW8 (mouse Liprin-α1) , Q8BSS9 (mouse Liprin-α2) and B8QI36 (mouse Liprin-α4).

April 2020

Field-specific reporting

Please select the one below that is the best fit for your research. If you are not sure, read the appropriate sections before making your selection.

☒ Life sciences ☐ Behavioural & social sciences ☐ Ecological, evolutionary & environmental sciences

For a reference copy of the document with all sections, see nature.com/documents/nr-reporting-summary-flat.pdf

Life sciences study design

All studies must disclose on these points even when the disclosure is negative.

Sample size	Sample size was determined based on the number of observations used in similar studies published in the field (examples: PMID27537483, PMID29606581, PMID32616470, PMID27537484, PMID17442248 or PMID31535974).
Data exclusions	All data that met quality standards (described in the methods section) were included. No outliers were excluded.
Replication	The number of replicates is reported in each figure. For cultured neurons, quantified data were acquired from at least three independent cultures, with the exception of electron microscopy (two independent cultures). Replications were successful and all replicates are included in the quantitative analyses.
Randomization	Data derived from animals were not randomized, but pooled by genotype and/or condition. Data collected in cell lines were not randomized, but pooled by protein expression and/or treatment.
Blinding	The experimenter was blinded throughout data collection and analyses for experiments in cultured neurons and cell lines that compare genotypes and/or drug treatments.

Reporting for specific materials, systems and methods

We require information from authors about some types of materials, experimental systems and methods used in many studies. Here, indicate whether each material, system or method listed is relevant to your study. If you are not sure if a list item applies to your research, read the appropriate section before selecting a response.

Materials & experimental systems

n/a	Involved in the study	
☐	☒	Antibodies
☐	☒	Eukaryotic cell lines
☒	☐	Palaeontology and archaeology
☐	☒	Animals and other organisms
☒	☐	Human research participants
☒	☐	Clinical data
☒	☐	Dual use research of concern

Methods

n/a	Involved in the study	
☒	☐	ChIP-seq
☒	☐	Flow cytometry
☒	☐	MRI-based neuroimaging

Antibodies

Antibodies used	The following primary antibodies were used: mouse anti-Bassoon (Enzo ADI-VAM-PS003-F), rabbit anti-Liprin-α1, -2, -3 & -4 (gifts from S. Schoch), rabbit anti-RIM (SySy 104003), mouse anti-PSD-95 (NeuroMab 73-028), mouse anti-Gephyrin (SySy 147021), mouse anti-Synapsin-1 (SySy 106001), guinea pig anti-Synaptophysin (SySy 101004), rabbit anti-RIM-BP2 (SySy 316103), rabbit anti-Munc13-1 (RRID: AB_887733), rabbit anti-CaV2.1 (SySy 152203), mouse anti-HA (Biolegend 90150), rabbit anti-MAP2 (SySy 188002), mouse anti-MAP2 (Sigma-Aldrich Cat# M4403), mouse anti-GluA1 (Sysy 182411), mouse anti-Synaptophysin (SySy 101011), and rabbit anti-Liprin-α3, anti-phospho-S760 Liprin-α3 and anti-phospho-S764 Liprin-α3 (custom-made as described in the manuscript).<br><br>The following secondary antibodies were used: goat anti-mouse IgG HRP-conjugated (MP Bio Cat# 0855550), goat anti-rabbit IgG HRP-conjugated (MP Bio Cat# 0855676), goat anti-rabbit IgG Alexa Fluor 488 (Thermo Fisher Scientific Cat# A-11034), goat anti-mouse IgG1 Alexa Fluor 488 (Thermo Fisher Scientific Cat# A-21121), goat anti-mouse IgG1 Alexa Fluor 555 (Thermo Fisher Scientific Cat# A-21127), goat anti-mouse IgG2a Alexa Fluor 555 (Thermo Fisher Scientific Cat# A-21137), goat anti-guinea pig IgG Alexa Fluor 633 (Thermo Fisher Scientific Cat# A-21105), and goat anti-mouse IgG Alexa Fluor 633 (Thermo Fisher Scientific Cat# A-21050).<br><br>RRIDs are provided in the manuscript when available.
Validation	The following antibodies essential to the key conclusion of this manuscript have been validated for immunostainings as performed in this study using mouse gene knockout as controls. RIM (SySy 140003) has been knockout-validated by PMID29606581. CaV2.1 (SySy 152203) has been knockout-validated by PMID32616470. Liprin-α2, -α3 and -α3 p760 antibodies are knockout-validated by this study. Munc13-1 (SySy 126 103) and RIM-BP2 (SySy 316 103) have been validated using genetic mutations that ablate these proteins through knockout of RIM+ELKS, described in PMID27537483.

The HA antibody (Biolegend 90150) is verified against controls that do not express an HA-tagged protein, both in this study and in previous experiments (PMID32616470).

The following antibodies (used as markers for immunostaining in this study) have been extensively used in immunostaining of mouse brain tissue. For PSD-95 (NeuroMab 73-028), see: PMID30396995, PMID31231192, PMID31601770, PMID31663850, PMID32616470. For Synaptophysin (SySy 101004), we refer to the manufacturer's webpage (https://sysy.com/product/101004) that states that this antibody has been assessed as specific for synaptophysin 1. For Gephyrin (SySy 147021), we refer to the manufacturer's webpage (https://sysy.com/product/147021) that states that this antibody has been assessed by the manufacturer as specific for the brain specific 93 kDa splice variant phosphorylated at Ser-270. For Bassoon (Enzo ADI-VAM-PS003-F), see PMID31988435, PMID32187536, PMID23267846, PMID19036990, PMID12163476, PMID12628184, PMID29606581, PMID: 32616470. For MAP2 (SySy 188002), we refer to the manufacturer's webpage (https://www.sysy.com/product/188002) that states that this antibody has been assessed by the manufacturer as specific for MAP2 (recognizes all four isoforms). For MAP2 (Sigma-Aldrich Cat# M4403), we refer to the manufacturer's webpage (https://www.sigmaaldrich.com/catalog/product/sigma/m4403?lang=en&region=US) that states that the antibody reacts with all known forms of MAP2, does not cross-react with other MAPs or tubulin, and shows selective labeling of dendritic trees throughout the brain.

Synaptophysin (SySy 101011), used for western blots, has been extensively used in mouse brain tissue. We refer to the manufacturer's webpage (https://sysy.com/product/101011), which states that this antibody has been assessed as specific for synaptophysin 1.

The remaining antibodies used in this study have been validated for immunostaining in mouse brain tissue. Liprin-α1 and –α4 (gifts from S. Schoch) have been reported before in mouse brain tissue (PMID21618221). For Synapsin-1 (SySy 106001), we refer to the manufacturer's webpage (https://sysy.com/product/106011) that states that this antibody has been assessed as specific for synapsin 1a and 1b independent of the phosphorylation state. For GluA1 (Sysy 182411), we refer to the manufacturer's webpage (https://sysy.com/product/182411) that states that this antibody detects GluA1, 2, and 3 in transfected cells and, due to sequence homology, it likely cross-reacts also with GluA4.

Eukaryotic cell lines

Policy information about cell lines

Cell line source(s)	HEK293T cells, purchased as identified cell line from ATCC, Cat#: CRL-3216, RRID:CVCL_0063.
Authentication	The cell line is sold as identified cell line from ATCC, but was not further autheticated after purchasing it.
Mycoplasma contamination	Purchased as mycoplasma free cell line from ATCC, no assessment of mycoplasma contamination was performed after purchasing the cells.
Commonly misidentified lines (See ICLAC register)	None.

Animals and other organisms

Policy information about studies involving animals; ARRIVE guidelines recommended for reporting animal research

Laboratory animals	Male and female mice were used in this study. Liprin-α2 mutant mice were maintained on a mixed background (C57BL/6 and 129/Sv). Liprin-α3 mutant mice were generated on a mixed background (FVB/NCrl and 129/Sv), and these two lines were crossed to each other. Controls were cultured neurons form the same mice infected with a different virus or cultured neurons from sibling mice of the experimental mice/neurons. All cultures were made from postnatal day 0 -1 mice. Mice were housed in a mouse facility room at 20-24 ºC (set point 22 ºC) and 35-70% (set point 50%) humidity on a regular dark/light cycle.
Wild animals	None.
Field-collected samples	None.
Ethics oversight	All animal experiments were approved by the Harvard University Animal Care and Use Committee.

Note that full information on the approval of the study protocol must also be provided in the manuscript.
