# Supplementary material for: PKC-phosphorylation of Liprin-α3 triggers phase separation and controls presynaptic active zone structure
Source: Nat Commun. 2021 May 24;12:3057. doi: 10.1038/s41467-021-23116-w (PMC8144191; doi:10.1038/s41467-021-23116-w)
Supplement: Supplementary file 6 — Source Data [file 41467_2021_23116_MOESM6_ESM.zip › Source Data Western.pdf]

Figure 2b

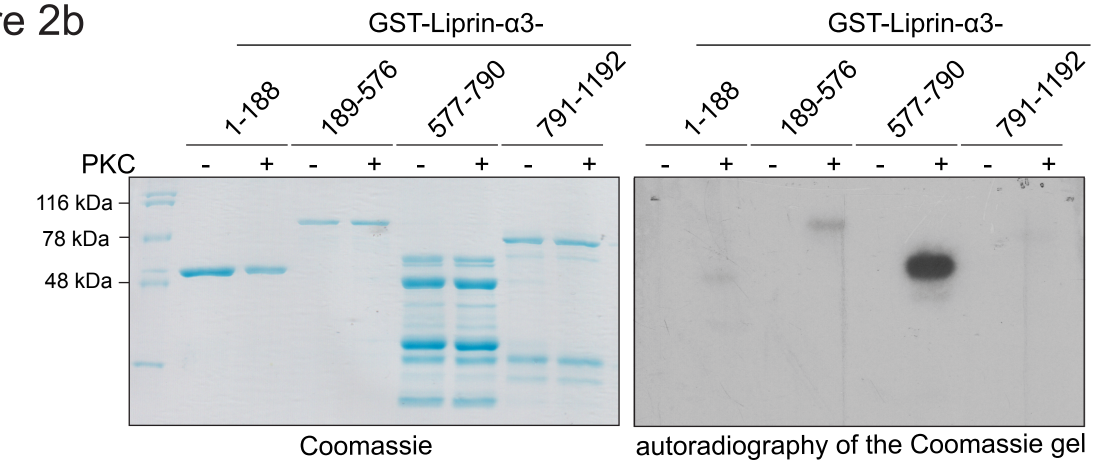

Figure 2c

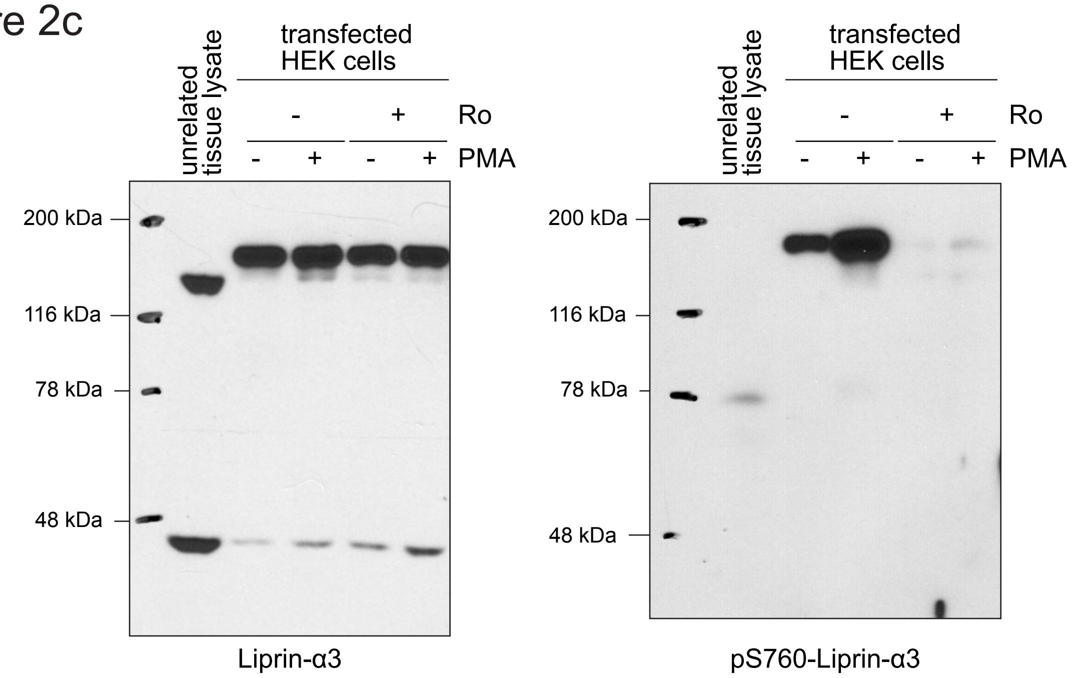

Figure 2d

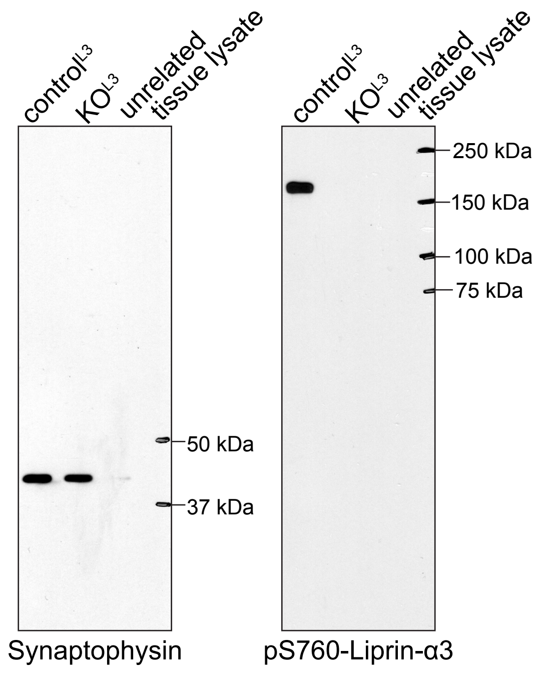

Supplementary Figure 2d

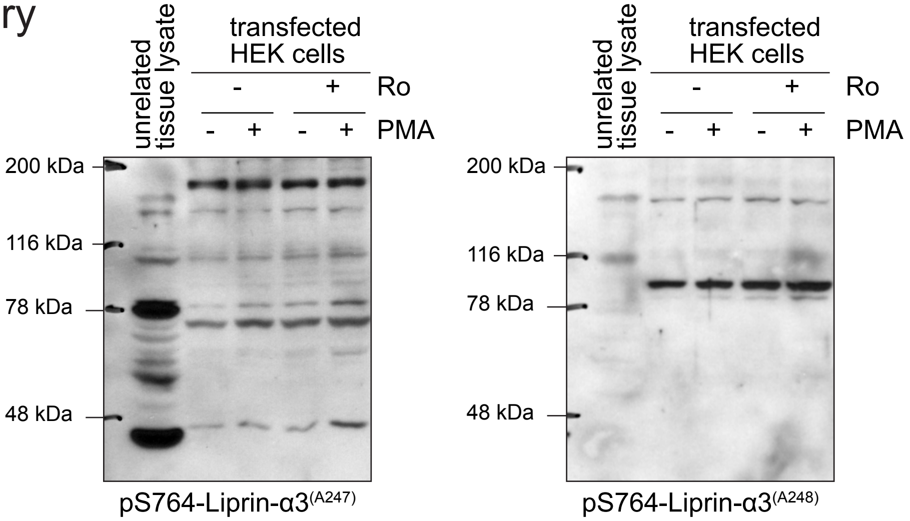

Supplementary Figure 2e

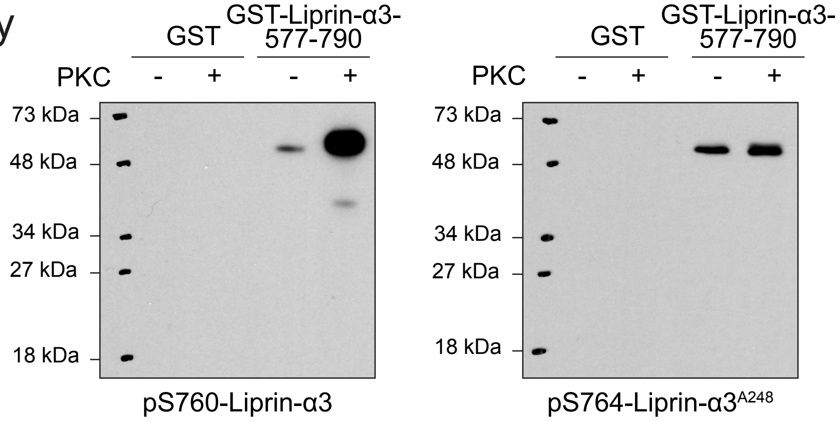

Supplementary Figure 2f

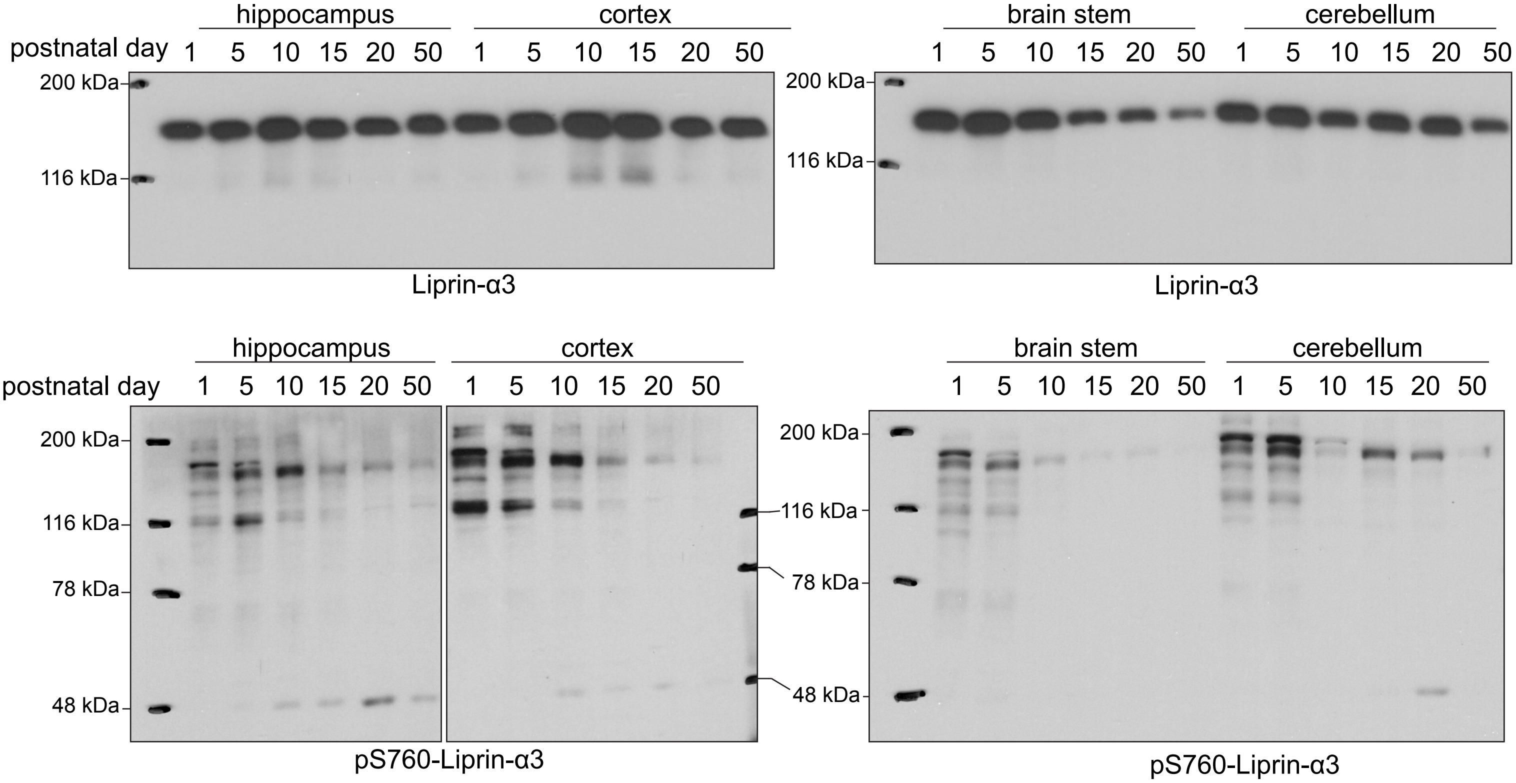

Supplementary Figure 8d

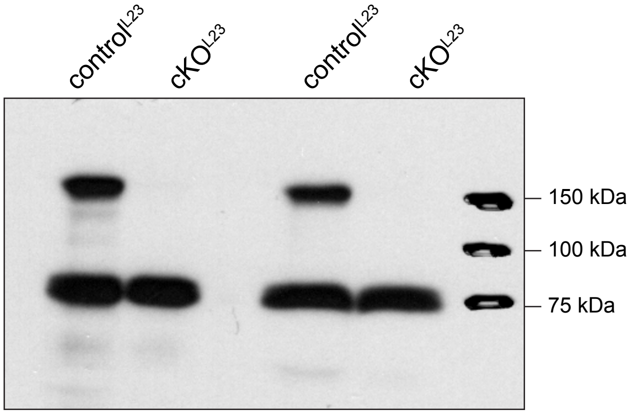

Liprin-α2 and Synapsin

Supplementary Figure 9a

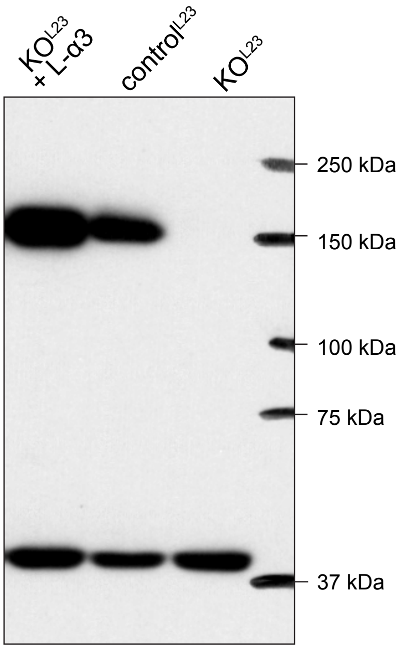

Liprin-α3 and Synaptophysin

Supplementary Figure 9i

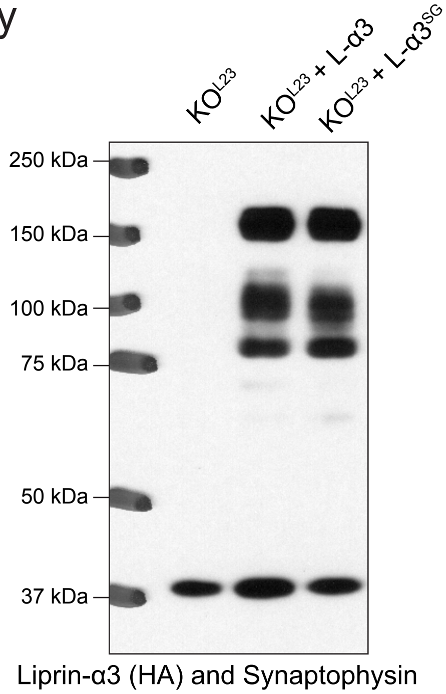

Liprin-α3 (HA) and Synaptophysin
